# Intraoral Microvascular Anastomosis in Immediate Free Flap Reconstruction for Midfacial Tumor Defects: A Retrospective Multicenter Study

**DOI:** 10.3390/jcm12227064

**Published:** 2023-11-13

**Authors:** Peer W. Kämmerer, Milad Tavakoli, Alexander Gaggl, Massimo Maranzano

**Affiliations:** 1 Department of Oral and Maxillofacial Surgery, Facial Plastic Surgery, University Medical Centre Mainz, Augustusplatz 2, 55131 Mainz, Germany; 2 Department of Oral and Maxillofacial Surgery and Facial Plastic Surgery, Manchester University NHS Foundation Trust (MFT), Manchester M13 9WL, UK; milad.tavakoli@mft.nhs.uk (M.T.); massimo.maranzano@manchester.ac.uk (M.M.); 3 Department of Oral and Craniomaxillofacial Surgery, Paracelsus Medical University, 5020 Salzburg, Austria; a.gaggl@salk.at

**Keywords:** free flap, midface reconstruction, intraoral anastomoses, reconstructive surgery, case series, facial vessels, malignant tumor

## Abstract

(1) Background: The current landscape of midface reconstruction is marked by ongoing evolution, with notable advancements in surgical techniques, microvascular procedures, and the implementation of multidisciplinary approaches, all of which have significantly enhanced both functional and aesthetic outcomes. Conventionally, microvascular anastomoses for free flaps in midfacial reconstruction have been executed using cervical vessels. However, this approach necessitates neck access, resulting in extraoral scars and a substantial pedicle length. In light of these considerations, using intraoral anastomoses via the facial vessels emerges as a promising alternative. This retrospective multicentric study aims to provide a comprehensive account of immediate midface reconstruction through intraoral anastomoses. (2) Methods: Between 2020 and 2023, patients were included who underwent intraoral resection of midface/orbit segments (Brown Classes I-VI) as a result of malignant diseases. In all cases, immediate reconstruction was accomplished by utilizing the facial vessels through an intraoral approach. Outcome criteria were identification of vessels, parotid duct or facial nerve damage, success of vascular anastomoses, and flap survival. (3) Results: A total of 117 patients with 132 flaps (91 osseous and 41 cutaneous) were included. The intraoral preparation of facial vessels was successfully completed in less than 1 h, and no complications related to the dissection or anastomoses were observed. In two cases, the vessel diameter was insufficient to facilitate anastomoses, necessitating adopting an extraoral approach. During a follow-up period of 48 months, two osseous flaps were lost, accounting for a 1.5% loss rate out of 132 flaps used. Additionally, 3 flaps experienced partial loss, including a skin island of a scapula, the border zone of a femur, and a rectus flap, resulting in a 2.3% partial loss rate out of 130 flaps utilized. (4) Conclusions: This case series underscores the feasibility of employing intraoral anastomoses for immediate complex midface reconstruction following oncological resection. This approach is particularly advantageous for flaps with shorter pedicles, as it helps mitigate external scarring and minimizes the risk of facial nerve injury.

## 1. Introduction

Reconstructing complex midface deformities following oncologic resections presents a formidable challenge. In such cases, vascularized free tissue transfers emerge as an excellent option. Though, these free tissue transfers, while invaluable in reconstructive surgery, are not without limitations. The primary drawbacks encompass prolonged operative times, potential donor site morbidity, the need for meticulous microsurgical skills, the risk of partial or total flap failure, and the possibility of postoperative complications, including thrombosis and infection. Furthermore, the availability of appropriate recipient vessels, as well as patient factors such as comorbidities and overall health, can influence the feasibility and success of these procedures. Despite these challenges, the benefits of vascularized free tissue transfers in complex reconstruction often outweigh the drawbacks, making them an indispensable tool in modern surgical practice. Especially in cases involving larger midfacial defects, autologous reconstruction proves advantageous, emphasizing a preference for local tissue whenever feasible. Additionally, the utilization of free flaps is considered when necessary. This approach may present benefits over prosthetic rehabilitation, where obturator prostheses are employed [1,2,3]. Alternative treatment options, like zygomatic implants, may present intriguing possibilities. However, it is important to note that the existing evidence in this context remains limited [4,5]. 

Given that oncologic treatment frequently necessitates concurrent regional lymph node management through procedures such as neck dissection, it ensures an adequate vascular access [6]. In the majority of cases, microvascular anastomoses are performed in the neck region, involving branches of the external carotid artery and the internal jugular vein. Conversely, in situations involving nasal and paranasal sinus malignancies and small maxillary oral squamous cell carcinomas (T1/T2 and/or a depth of invasion < 3 mm, with negative sentinel node biopsy), the risk of lymph node involvement is low. In such cases, elective neck dissection may not be deemed necessary in the absence of nodal involvement [7,8,9]. In these cases, additional cervical access is linked to elevated morbidity rates. Moreover, the extraoral vessel approach can pose challenges in terms of accessing the tumor site during the reconstruction process. This challenge is particularly pronounced when dealing with flaps characterized by a short pedicle and when the course of the pedicle from the neck to the reconstructed site is compromised [10]. In instances involving flaps with a short pedicle and requiring extraoral anastomosis, techniques for pedicle elongation, such as interposition vein grafting, become necessary. However, it is essential to recognize that this procedure carries specific risks that can impact the flap’s overall viability and success [11,12].

The use of intraoral anastomoses with facial vessels has been acknowledged as a promising alternative approach to address concerns associated with cervical scarring and challenges related to pedicle length [10,13]. A potential limitation of this approach lies in the less predictable variability and the tortuosity of the facial vessels within the cheek [14,15]. Therefore, it is advisable to visualize these vessels, at the very least, using Doppler ultrasonography before proceeding with surgery. Furthermore, scars in the vestibular mucosa have been associated with a more intricate process of vessel preparation [12,13]. It is important to consider that patients with limited mouth opening and/or temporomandibular joint dysfunction may not be ideal candidates for this approach [16,17]. Dissecting the vessels in this region, particularly the vein, can be challenging, and the buccal vessels may not provide adequate flow for successful anastomosis. Hence, it is imperative to discuss alternative techniques of extraoral anastomosis with the patient [18]. 

From a recent literature review, 122 patients with maxillofacial defects were treated between 2009 and 2021 with flaps nourished by intraoral anastomoses [12]. Nonetheless, it is worth noting that the existing literature on intraoral anastomoses of free flaps primarily emphasizes secondary reconstruction and includes a substantial number of patients with benign lesions [10,13,16,19,20]. It is important to highlight that there is a limited body of the literature consisting of only one case series that explores the use of intraoral anastomoses for immediate reconstruction of maxillary defects following oncological excisions. This series encompassed a total of six patients, with only two of them having a history of malignant diseases [21]. As a result, the objective of this retrospective multicentric analysis was to substantiate the feasibility of immediate midface reconstruction following oncologic resection by employing free flaps and intraoral anastomoses within a larger and more diverse patient cohort.

## 2. Materials and Methods

The indications for intraoral anastomoses in this study primarily revolved around tumor defects affecting the skull base and midface region. Patients retrospectively included in the study in the period between January 2020 and January 2023 had malignant tumors of the midface, but the need for neck access was deemed unnecessary. This applied to situations involving nasal and paranasal sinus malignancies and small maxillary oral squamous cell carcinomas (T1/T2 and/or a depth of invasion <3 mm), with confirmed negative sentinel node biopsy results. Each defect was classified in accordance with the Brown and Shaw classification system [22]. This system ranges from I to VI and is a well-established method to categorize defects of the midface based on their size, location, and complexity. In brief, it differentiates between limited defects of the maxilla without oronasal fistula (Brown Class I), extended defects of the maxilla not involving the orbit (Brown Class II), extended defects of the maxilla involving the periorbital area or with the orbital floor (Brown Class III), extended maxillary defects with orbital enucleation or exenteration (Brown Class IV), orbitomaxillary defects (Brown Class V), and nasomaxillary defects (Brown Class VI).

Exclusion criteria were patients not suitable for vascularized free tissue transfer, e.g., with poor general health, vascular insufficiency, or the absence of appropriate donor sites. The decision for or against free flap transfer was always made in consultation with a multidisciplinary team, including the surgeon, anesthesiologist, and other relevant specialists. The choice of flap selection was determined by the individual surgeon’s clinical judgment, considering the unique characteristics of the patient’s defects and the availability of an appropriate donor site. Patient data for this study were collected from multiple sources, including the University Medical Center Mainz, Germany, under the supervision of surgeon PWK; the Paracelsus Medical University, Austria, led by surgeon AG; and the Manchester University NHS Foundation Trust, UK, where surgeons MT and MM were involved. The study adhered to the principles outlined in the Declaration of Helsinki and received approval from the Institutional Review Board of Rhineland-Palatinate (2019-14678).

### Perioperative Assessment

In all instances, a preoperative ultrasound Doppler examination of the facial/labial artery and the angular/facial vein was performed. This evaluation was subsequently repeated during the surgical procedure using a sterile Doppler probe. Doppler probes for the assessment of vessel flow involves a systematic approach. To ensure accurate measurements, an appropriate amount of conductive gel or coupling agent should be applied, facilitating acoustic coupling between the Doppler probe and the skin. The probe must be meticulously positioned to achieve the optimal angle and orientation for precise vessel interrogation, while maintaining consistent gentle pressure to prevent vessel compression. Movements should be deliberate and unhurried to capture the full spectrum of blood flow velocities. Interpretation of Doppler signals is based on their audible or visual characteristics; high-pitched continuous sounds typically denote rapid flow, whereas low-pitched intermittent sounds signify slower flow.

**Figure 1:**
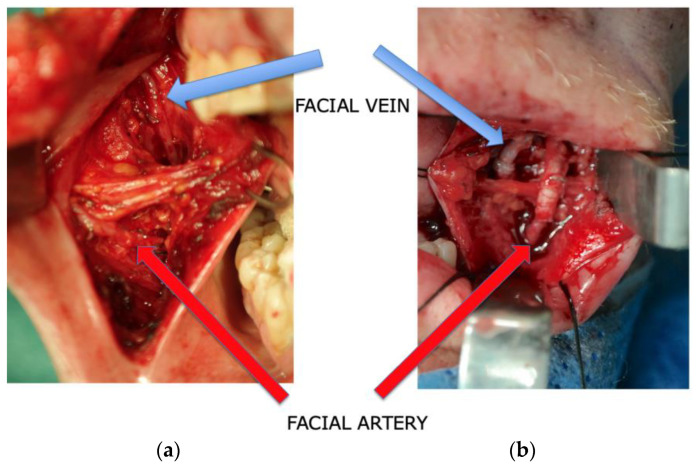
Identification of facial vessels (**a**) and after conducting intraoral anastomoses (**b**).

**Figure 2:**
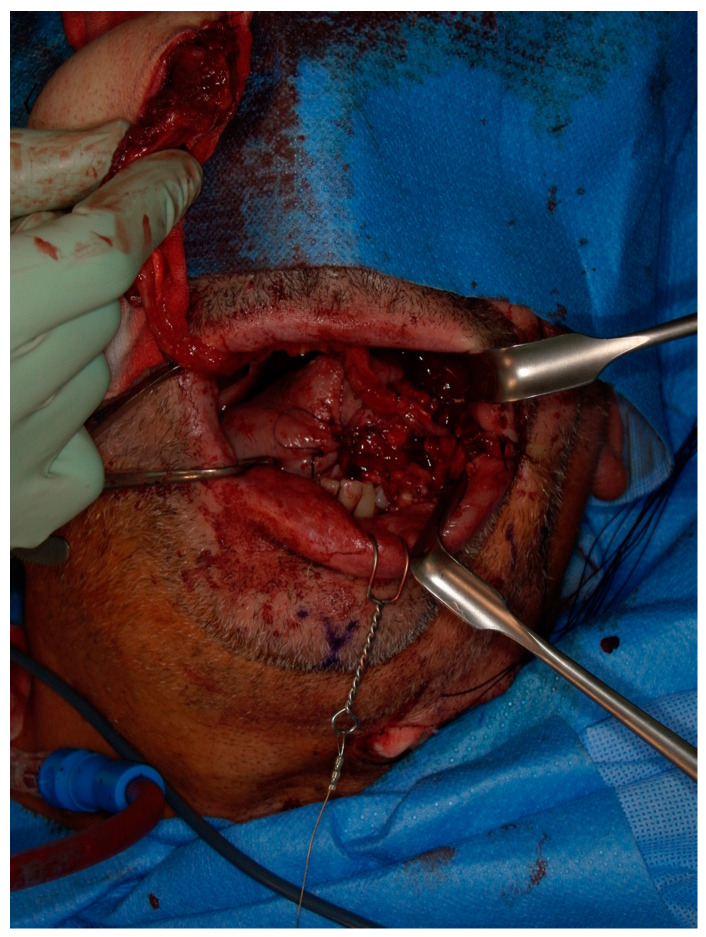
ALT flap for reconstruction Class I defect of the left maxilla after anastomosis.

Subsequently, the facial vessels were meticulously identified prior to tumor resection, employing an intraoral V-shaped incision during the surgical procedure. This incision, measuring at least 2–4 cm in length, was precisely placed directly anterior–inferior to Stensen’s duct. The dissection was extended through the buccinator muscle, ensuring careful preservation of facial nerve branches running laterally to the artery. With a strong emphasis on meticulous blunt dissection, these nerve branches were safeguarded. Following this preparatory phase and with thorough protection of the parotid gland duct and facial nerve, a vascular dissection spanning 2 to 4 cm was carried out in a retrograde fashion. The primary objective of this dissection was to attain an adequate vessel diameter to facilitate subsequent anastomoses. Notably, the facial vein was consistently situated in the posterior and medial vicinity of the artery, predominantly within the buccal fat pad. Its terminal point was consistently identified at the thyrolingual trunk, with the mean distance between the artery and vein measuring approximately 22 mm [23], all within the depths of the buccal fat pad [21] (Figure 1a,b and Figure 2). This early stage vessel dissection was undertaken with a particular rationale: to capitalize on the optimal functionality of the vessels at this juncture. This approach, particularly advantageous for the vein, minimizes the risk of vessel collapse and contributes to the overall ease of the dissection process.

**Figure 3:**
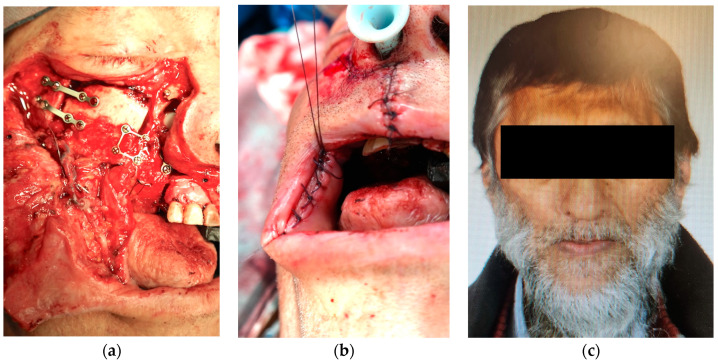
A case involving the reconstruction of the right maxilla using a DCIA (deep circumflex iliac artery) flap (**a**). Subsequently, (**b**) showcases the patient’s condition following wound closure. In (**c**), the final result is depicted, with the image provided by the patient.

Following tumor resection and the parallel elevation of the flap, employing a two-team approach whenever possible, anastomoses were typically conducted at the angle of the mouth. The arterial anastomosis was performed using non-resorbable monofilament sutures (8-0, 9-0, or 10-0, Ethilon, Johnson & Johnson, New Brunswick, NJ, USA). For venous anastomoses, the choice was between a coupler device (QuaMedical, Zuidwolde, The Netherlands) or non-resorbable monofilament sutures (8-0, 9-0, or 10-0, Ethilon, Johnson & Johnson). Primarily, end-to-end anastomoses were the preferred technique, with the diameters of the facial vessels typically matching those of the donor vessel pedicle (Figure 3a–c).

Following the successful anastomosis, specific postoperative measures were implemented in select cases. These measures included the insertion of a silicone drain for wound drainage. Additionally, an implantable Doppler probe was positioned on the artery distal to the anastomosis for monitoring purposes. The mucosa was then sutured using absorbable material (Vicryl, Ethicon Inc, Bridgewater, NJ, USA).

Postoperatively, the flaps were subjected to clinical monitoring, following the previously established protocol. In essence, this monitoring encompassed the assessment and documentation of various clinical parameters related to flap perfusion, which included observations of color, temperature, re-capillarization time, and tissue turgor. Additionally, if an implantable Doppler probe had been inserted, the probe’s signal was consistently recorded. To facilitate the monitoring process, specific point values were assigned to each clinical category (e.g., color, temperature, re-capillarization time, turgor). Surgical re-exploration criteria were considered met if the total point value equaled or exceeded 9 or if two variables scored 6 points. Further, re-exploration was also indicated if the flap’s color remained pale white or blue for over 60 min and the re-capillarization time was either not detectable or less than 1 s [24]. Postoperative monitoring was conducted at 2-h intervals during the initial 24 h, followed by 4-h intervals during the subsequent 24 h after surgery. All patients received perioperative antibiotics for at least 72 h with a combination of amoxicillin and sulbactam or clavulanic acid or clindamycin in cases of penicillin allergy. An i.v. bolus of 5000 IU heparin was administered 5 min before the flap disconnection from the donor site; further antithrombotic protocol consisted of a low dose of enoxaparin–sodium subcutaneously (adjusted for weight and kidney function) for at least six days postoperatively, starting 12 h after the end of the surgical procedure.

## 3. Results

The comprehensive retrospective study encompassed a total of 117 patients, consisting of 77 males and 40 females, with an average age of 60.2 years. Each defect was categorized in accordance with the Brown and Shaw classification system [22], yielding the following classifications:

1. Limited defects of the maxilla without oronasal fistula (Brown Class I; n = 26);
2. Extended defects of the maxilla not involving the orbit (Brown Class II; n = 19);
3. Extended defects of the maxilla involving the periorbital area or with the orbital floor (Brown Class III; n = 10);
4. Extended maxillary defects with orbital enucleation or exenteration (Brown Class IV; n = 14);
5. Orbitomaxillary defects (Brown Class V; n = 21);
6. Nasomaxillary defects (Brown Class VI; n = 27).

To address these diverse defect types, a wide array of flaps was utilized for reconstruction, including:

1. Osteomyocutaneous medial femoral condyle flaps (n = 44),
2. DCIA flaps (n = 34);
3. Myocutaneous radial forearm flaps (n = 8);
4. Myocutaneous ulnar forearm flaps (n = 7);
5. Osteomyocutaneous fibula flaps (n = 7);
6. Osteomyocutaneous scapula flaps (n = 6);
7. Myocutaneous radial-thenar flaps (n = 6);
8. Myocutaneous rectus flaps (n = 5);
9. Myocutaneous latissimus dorsi flaps (n = 5);
10. Myocutaneous ALT flaps (n = 4);
11. Myocutaneous buccal flaps (n = 3);
12. Myocutaneous lateral arm flaps (n = 2);
13. Myocutaneous peroneus flaps.

It is noteworthy that, in 15 out of 117 cases, the complexity of the defects necessitated the use of two flaps (Table 1). As a wide range of different flap types were utilized in this study, detailed information on flap harvesting and defect reconstruction has not been included in this paper. Readers are encouraged to refer to the relevant literature for comprehensive insights on these specific topics.

Tumor resection with clear margins was successfully accomplished in all cases. The identification of facial vessels through palpation and intraoral Doppler ultrasound proved to be straightforward and unproblematic for every case. Notably, the mean duration for the intraoral preparation of the facial vessels was less than one hour, and no complications were encountered during this phase. Furthermore, there were no instances of parotid duct damage or facial nerve palsy. Additionally, no instances of intraoral wound dehiscence were observed.

### Microvascular Reconstruction

In a single case, the facial artery was found to be too diminutive, and in another case, the facial vein exhibited insufficient size for anastomosis, with both vessels measuring less than 1 mm in diameter (2/132; 1.5%). In these particular patients, anastomoses were carried out at the ipsilateral neck region, utilizing the thyroid artery and the internal jugular vein.

**Figure 4:**
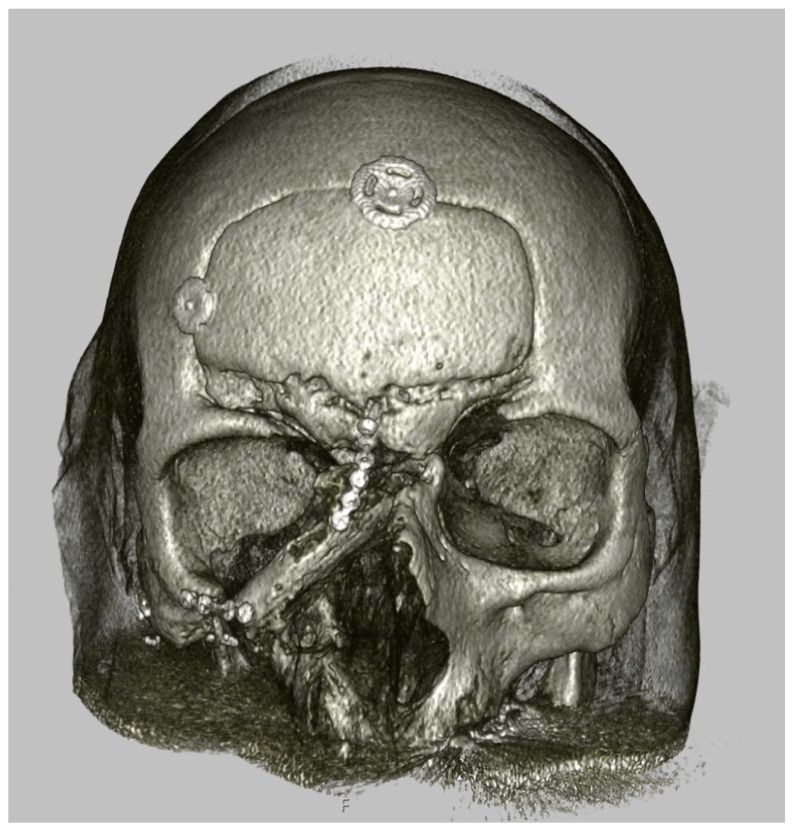
A 3D overview of the CT dataset reveals a Class V defect resulting from osteosarcoma, which was successfully reconstructed using a scapula flap with intraoral anastomoses. This reconstruction was assessed at the 36-month postoperative mark. Notably, in this specific case, the skin island was lost seven days after the surgical procedure.

In 30 cases, a Cook–Swartz Doppler probe (Cook Medical LLC, Bloomington, IN, USA) was surgically implanted on the artery chosen for flap monitoring. These probes were typically removed after a duration of 4 to 7 days. [25]. For eight flaps, hyperspectral imaging was used in addition to clinical monitoring. The mean follow-up duration was 48 months. Within this timeframe, two cases experienced complete flap failure, occurring at 3 and 56 days after surgery (2/132; 1.5%). Notably, the first case had a prior history of irradiation at the surgical site, and the flap failure transpired following radiotherapy in the second case. Additionally, partial flap failure was observed in three cases, with occurrences 4 days after surgery (involving partial soft tissue necrosis at the border zone of an osteomyocutaneous medial femoral condyle flap), 7 days after surgery (involving the skin island of the scapula flap, as depicted in Figure 4), and 15 days after surgery (involving the skin of the rectus muscle). These instances accounted for 3 out of 130 cases (2.3%). In response, these cases underwent revision and intraoral re-anastomosis.

All patients in the study attained satisfactory functional and aesthetic outcomes following the reconstructive procedures. In four cases, where only soft tissue coverage of Class II and III defects was initially performed (two radial forearm flaps and two ulnar forearm flaps), a subsequent second-stage procedure involved the placement of zygomatic implants to provide support for a dental prosthesis.

## 4. Discussion

The reconstruction of midface deformities and malignancies often capitalizes on the access routes established during tumor resection or as a result of traumatic or malignant events. This access enables the implementation of extraoral microvascular anastomosis techniques. In cases where conventional access routes are unavailable, alternative approaches may be considered. These alternatives encompass access through the nasolabial fold [26] and submandibular or preauricular approaches [27,28]. The intraoral microvascular anastomosis technique was initially documented by Givol et al. in 1998, with its inaugural application observed in two cases involving anterior mandibular reconstruction [29]. Subsequently, Gaggl et al. published their findings, which encompassed the use of nine osteocutaneous free flaps for secondary reconstruction of substantial maxillary or mandibular defects. The authors reported no instances of flap loss and noted the absence of both intraoperative and postoperative complications [13]. Following these initial observations, subsequent reports surfaced in the literature detailing the utilization of intraoral anastomosis techniques in an array of clinical scenarios. These included cases involving cleft palate, trauma, atrophy, benign tumors, cystectomies, decortication following osteomyelitis, and fibrous dysplasia [10,17,18,30]. 

Within the confines of the current case series, which stands as the most extensive collective reported thus far for postablative primary reconstruction in cancer patients, it has been underscored that the intraoral anastomosis technique holds promise for immediate craniomaxillary reconstruction cases where concurrent neck treatment is not indicated. Beyond the advantages of avoiding supplementary skin incisions, this approach also presents potential benefits concerning the preservation of facial nerve branches. These branches tend to course beneath the platysma and lateral to the facial vessels, obviating the need for transposition in the context of the intraoral approach [13]. Moreover, the intraoral anastomosis technique offers distinct advantages due to its proximity to the resection site, which can be particularly beneficial when pedicle length and positioning are critical considerations. Nonetheless, it is essential to note that this technique does present challenges. The initial opening phase of the anastomosis and potential bleeding have been reported as less easily controlled, necessitating a high degree of microsurgical expertise [17]. Furthermore, it is imperative to acknowledge the considerable variability in the size of the facial vessels, underscoring the importance of meticulous pre- and intraoperative assessment. Typically, this assessment involves the use of a Doppler device in conjunction with clinical palpation of the pulse on the inferior aspect of the mandible. Calva et al. conducted an investigation involving 20 hemifacial dissections to explore the relationship of the facial artery with various topographic landmarks. Their findings revealed that, on average, the facial artery is located at a mean distance of 93 mm from the lateral canthus, 19 mm from the oral commissure, 31 mm from the mandibular angle, and 18 mm from the papilla of Stensen’s duct [31]. Nonetheless, the course of the facial vein can add complexity to the dissection process. As demonstrated in the present study, there were instances where intraoral anastomoses were not feasible due to the small diameter of either the artery or vein, which could not be dissected adequately over the required 3–4 cm. Qui et al. also reported their experiences, noting that intraoral anastomoses were achievable in 9 out of 11 cases, with 2 of those cases necessitating revision and re-anastomosis. The authors utilized the facial artery in seven cases and the superior labial artery in two cases. Similar to our findings, one case in their report encountered a failure of intraoral anastomosis due to the small caliber of the recipient vessel, necessitating the use of extraoral anastomosis. In another instance, a DCIA flap experienced thrombosis one day after surgery [19].

It is noteworthy that, in this study, there were no instances where it took more than one hour to identify and prepare the recipient vessels. These findings align with the observations made in Gaggl’s study [13] and might pose a significant advantage over extraoral anastomoses. Conversely, it is important to acknowledge that the preparation of vessels through an intraoral approach has demonstrated greater complexity compared with the conventional method [12]. Moreover, it is essential to consider that in cases of secondary reconstruction following prior neck dissection, the facial vessels may have been ligated, rendering intraoral anastomosis unfeasible in such scenarios. 

The authors of this analysis contend that the study brings forth several key points. Firstly, it underscores that intraoral anastomosis techniques represent a feasible and effective approach for immediate reconstruction following midface malignancy-related procedures. Notably, this constitutes the third case series in the existing literature where intraoral anastomosis techniques have been employed for midfacial reconstruction in the context of malignancies. One of the previously reported case series focused on five palatal defects stemming from various causes, primarily involving secondary reconstruction [16]. In another case series, two out of six patients were diagnosed with malignant diseases and underwent immediate reconstruction using a medial femoral condyle flap and a fibula flap [21]. Drawing from the results of both prior case series and the present study, the authors wish to extend their recommendation that the intraoral anastomosis technique can be confidently applied in the immediate reconstruction of various tumor-related defects. Specifically, this approach is deemed suitable for addressing defects impacting the skull base, orbital region (Class V), combined maxillary and orbital defects (Class IV), maxillary defects (Classes I, II, III), as well as nose defects (Class VI) [22]. Continuing the discussion, the second point pertains to the instances of failure observed in the case series. Notably, two total failures were identified, both occurring in patients who had undergone radiotherapy. Additionally, there were three cases of partial failure, involving the skin of a medial femur, a rectus, and a scapula flap, necessitating revision of the anastomoses. Importantly, it is worth highlighting that these failures were not linked to the anastomosis technique itself, but rather the postoperative complications observed were consistent with those commonly encountered in conventional free flap procedures [32,33]. Furthermore, in line with the findings in the existing literature, it appears that intraoral anastomosis techniques exhibit suitability for a wide range of free flaps, without any distinct preferences among them [10,12]. In the remaining 127 cases, a high rate of success was observed using a diverse range of free flaps. However, it is important to examine two key considerations. Firstly, it is noteworthy that the majority of successful cases reported in the literature are typically associated with defects that did not result from malignancies, and often involved secondary reconstruction [10,13,18,30]. It is well-documented in the literature that the stage of the tumor can indeed influence the rate of flap failure [34]. Consequently, it can be cautiously deduced that intraoral microvascular anastomosis is not exempt from the challenges posed by oncological reconstruction, which is especially evident in patients experiencing total failure after radiotherapy. The second issue is rooted in the dearth of the comprehensive literature on the subject and raises a pertinent question: what are the contraindications for intraoral anastomosis? It is noteworthy that none of the cases in this study exhibited failure attributable to the anastomotic technique. It is essential to acknowledge that the data presented here are based on retrospective analysis of a single cohort of patients. Consequently, there is a critical need for prospective comparative studies to provide more robust evidence. As the use of this technique continues to gain prevalence in head and neck units both nationally and internationally, it becomes imperative to conduct comprehensive investigations into its limitations and advantages. In light of these considerations, the authors advocate for expanded research efforts and the comprehensive reporting of cases involving intraoral microvascular anastomosis. This endeavor is essential for the development of well-defined guidelines in the future, enhancing the understanding and application of this technique in clinical practice.

## 5. Conclusions

Collectively, with insights derived from the existing body of literature, this report presents promising outcomes when employing an intraoral approach for vessel anastomoses in immediate free flap reconstruction of the midface postoncologic resection. A notable advantage of this technique is its ability to circumvent the need for an extraoral cervical incision, consequently mitigating the issue of scarring. Additionally, this approach aids in preventing facial nerve damage and permits the utilization of short pedicle flaps for midface reconstruction. It is worth noting that an intraoral anastomosis procedure remains feasible even when a neck dissection is part of the surgical plan; in such cases, preserving the facial artery and vein is crucial. This preservation obviates the requirement for a vein graft in instances where the reconstruction flap’s pedicle is of insufficient length to reach the neck. Contrary to the advantages this technique offers, it is important to recognize its limitations. These are primarily associated with the unpredictability in the course of the facial vessels, including their varying diameters. Furthermore, it is not advisable to employ this technique in cases where patients have vestibular scars, restricted mouth opening, and/or temporomandibular joint dysfunction. Subsequently, a logical next step involves comparing extraoral standard anastomosis techniques concerning surgical time, success rates, and other patient-related outcomes.

## Figures and Tables

**Table 1:** Summary of the cases (F = female, M = male, ALT = anterior lateral thigh flap, RFFF = radial forearm flap, SUBSC = free flaps from the subscapular system, LAT ARM = lateral arm flap, ULNAR = ulnar free flap, DCIA = deep circumflex iliac artery bone flap, FIB = free fibula flap, FEMUR = medial femoral condyle flap, BUCCAL = myocutaneous buccal flap, RECTUS = rectus flap, THEN = radial thenar flap, PER = myocutaneous peroneus flap, MULTIPLE = more than one flap was used).

Class	Total	F	M	ALT	RFFF	SUBSC	LAT ARM	ULNAR	DCIA	FIB	FEMUR	BUCCAL	RECTUS	THEN	PER	MULTIPLE
I	26	7	19	1	5	0	2	6	2	1	6	0	0	3	0	0
II	19	7	12	1	1	0	0	1	12	1	0	0	0	3	0	0
III	10	5	5	0	0	0	0	0	3	5	0	0	5	0	1	4
IV	14	4	10	0	0	5	0	0	13	0	3	0	0	0	0	7
V	21	8	13	2	2	5	0	0	4	0	8	0	0	0	0	0
VI	27	9	18	0	0	1	0	0	0	0	27	3	0	0	0	4

## Data Availability

Data are available upon request from the authors.
